# Temporal dynamics of the early immune response following *Mycobacterium bovis* infection of cattle

**DOI:** 10.1038/s41598-024-52314-x

**Published:** 2024-01-31

**Authors:** Thomas Holder, Sreenidhi Srinivasan, Adrian McGoldrick, Gareth A. Williams, Simonette Palmer, John Clarke, Amanda O’Brien, Andrew J. K. Conlan, Nick Juleff, H. Martin Vordermeier, Gareth J. Jones, Vivek Kapur

**Affiliations:** 1 Animal and Plant Health Agency, Bacteriology, Addlestone, UK; 2 The Huck Institutes of Life Sciences, The Pennsylvania State University, University Park, PA USA; 3 Enfer Scientific, Unit T, M7 Business Park, Newhall, Naas, County Kildare Ireland; 4 Disease Dynamics Unit, Department of Veterinary Medicine, University of Cambridge, Cambridge, UK; 5 The Bill & Melinda Gates Foundation, Seattle, WA USA; 6 Department of Animal Science, The Pennsylvania State University, University Park, PA USA

**Keywords:** Immunology, Cellular immunity, Humoral immunity, Infectious diseases, Tuberculosis

## Abstract

Bovine tuberculosis is an infectious disease of global significance that remains endemic in many countries. *Mycobacterium bovis* infection in cattle is characterized by a cell-mediated immune response (CMI) that precedes humoral responses, however the timing and trajectories of CMI and antibody responses determined by newer generation assays remain undefined. Here we used defined-antigen interferon-gamma release assays (IGRA) and an eleven-antigen multiplex ELISA (Enferplex TB test) alongside traditional tuberculin-based IGRA and IDEXX *M. bovis* antibody tests to assess immune trajectories following experimental *M. bovis* infection of cattle*.* The results show CMI responses developed as early as two-weeks post-infection, with all infected cattle testing positive three weeks post-infection. Interestingly, 6 of 8 infected animals were serologically positive with the Enferplex TB assay as early as 4 weeks post-infection. As expected, application of the tuberculin skin test enhanced subsequent serological reactivity. Infrequent *M. bovis* faecal shedding was observed but was uncorrelated with observed immune trajectories. Together, the results show that early antibody responses to *M. bovis* infection are detectable in some individuals and highlight an urgent need to identify biomarkers that better predict infection outcomes, particularly for application in low-and-middle income countries where test-and-slaughter based control methods are largely unfeasible.

## Introduction

Bovine tuberculosis (bTB) is a chronic infection of livestock including cattle, buffalo, sheep and goats caused by members of the *Mycobacterium tuberculosis* complex (MTBC). This disease is of global economic impact and a threat to public health ^1–4^. Whilst the causative agent in countries like the USA, UK, or Ireland is almost exclusively *Mycobacterium bovis* (*M. bovis*), other members of the MTBC such as *M. caprae* or *M. orygis* can be the dominant cause of bTB in other geographies ^5,6^. The control and elimination of bTB is largely based on testing of animals using the tuberculin skin test that measures a type IV delayed-hypersensitivity response to MTBC exposure, rather than infection, followed by the removal (generally via slaughter) of reactor animals. Effective control is therefore dependent on the ability of ante-mortem diagnostics to identify infected animals with high sensitivity and specificity prior to them becoming infectious.

Based on experimental and natural infections of cattle, Pollock and Neill proposed a model of the temporal dynamics of anti-tuberculous immune responses in cattle (Neill, Pollock, modified and updated by De la Rua et al.) ^7,8^. They proposed that cell-mediated immune (CMI) responses such as those measured by tuberculin skin tests or interferon-gamma release assays (IGRA), developed relatively quickly (~ 3 to 6 weeks) post-infection, followed by the development of humoral immune responses to *M. bovis* antigens such as MPB83 ^9^. It is also well-established that application of the tuberculin skin test increases humoral immune responses (“anamnestic” responses, reviewed in Lyashchenko et al.), ^10^ and that serological assays for bovine tuberculosis require an anamnestic boost to realise their full performance potential. Furthermore, it has been hypothesised that during the later phases of disease progression in naturally infected animals, CMI responses contract while humoral responses are being maintained, resulting in what is called the “anergy” phase ^8,11^. However, this anergic phenotype is difficult to reproduce under experimental conditions, likely due to the long observation periods required to reach such an anergic state. Thus, given the early development of CMI responses after infection, tests based on measuring CMI responses (e.g. tuberculin skin test or IGRA) remain the cornerstone of diagnostic testing for bTB. In contrast, prior generations of serological assays have been less utilised due to the delayed onset of humoral responses and the requirement of a previous tuberculin skin test to improve case detection rates ^12^. However, the immunological response trajectories following *M. bovis* infection of cattle have not been assessed in the context of newer generations of serological or T-cell assays. Hence, we here assessed the temporal development of immunological responses of calves experimentally infected with *M. bovis* using a recently developed molecularly defined interferon-gamma release assay (IGRA) based on the antigens ESAT-6, CFP-10, and Rv3615c (DST), and an eleven-antigen based indirect chemiluminescent multiplex ELISA (Enferplex TB assay). Our results confirm the temporal precedence of IGRA over serological assays, but also show that a subset of animals (3 of 8) were serologically positive as early as 3 weeks post-infection with the Enferplex high sensitivity assay (Enfer Se), and increasing to 6 of 8 animals positive by week 4. Together, these results refine our understanding of the immune trajectories of *M. bovis* infection in cattle and highlight an urgent need to identify the next generation of biomarkers that better predict infection and disease outcomes.

## Results

### Cell-mediated immune response in experimentally *M. bovis-*infected animals

#### Interferon-gamma response

**Figure 1:**
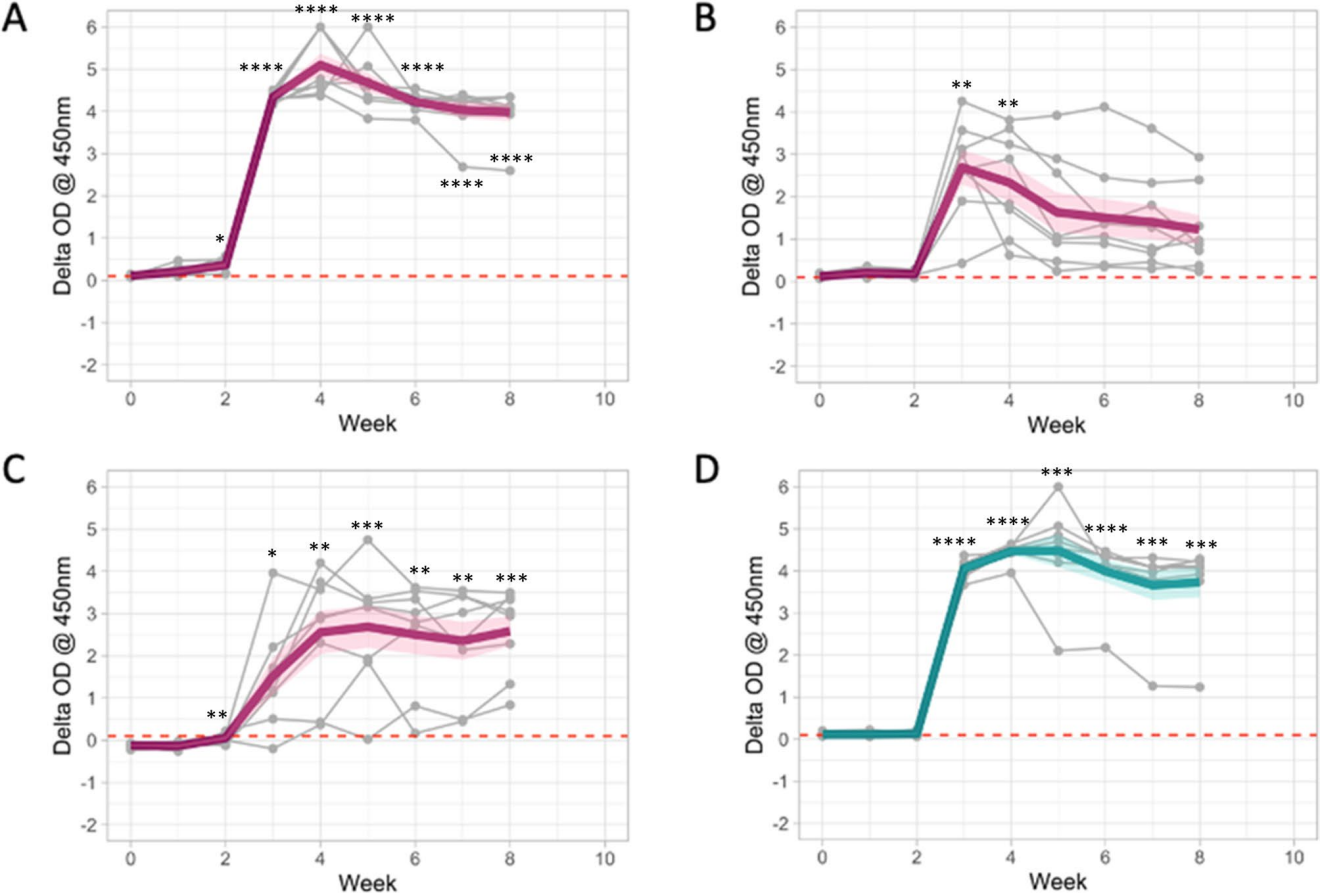
CMI-based Interferon-gamma response (*n* = 8). Blood samples from eight calves were taken both prior to and after experimental infection with *M. bovis* for use in whole blood IGRA using tuberculin and DST-P reagents. The background-corrected (delta) optical density (OD) values are plotted. Time is shown as weeks post-infection in the x-axis. (**A**) PPD-B; (**B**) PPD-A; (**C**) PPD(B-A); (**D**) DST-P. Responses of individual animals are shown (thin grey lines), as well as a trend line (thick line) that displays the sample mean (across animals) for each time point and 1 standard error on the mean (shaded area). Dashed horizontal red line: cut-off value for positivity at 0.1. *p < 0.05, **p < 0.01, ***p < 0.001, ****p < 0.0001, ANOVA with Dunnett’s multiple comparisons test (compared to the pre-challenge values).

The CMI triggered interferon-gamma response was assessed in experimentally infected animals using the commercial BOVIGAM™ ELISA with PPDs and DST-P (a cocktail of 13 peptides representing three antigens (ESAT-6, CFP-10 and Rv3615c)) used for in vitro stimulation. The schedule for testing is summarised in Table 1. Results are expressed as background-corrected optical density (OD) values of PPD-A, PPD-B, PPD(B-A) and DST-P (Fig. 1; supplemental Table S1). Although IGRA is generally interpreted as PPD(B-A), individual PPD-A and B trends post-infection are also shown. IGRA responses were noted as early as 2 weeks post-infection (Fig. 1), with all animals testing positive (response ≥ 0.1 OD cut-off value) with PPD(B-A) and DST-P at week 4 post-infection. The animals remained IGRA positive for the rest of the trial duration, with one exception (animal ID 1642) that dropped below cut-off for PPD(B-A) at week 5. At week 4, the peak response was attained for DST-P with an observed mean (± standard deviation) IFN-γ OD response of 4.3 ± 0.3, while PPD (B-A) IFN-γ response peaked at week 5 with mean responses of 2.7 ± 1.4. Importantly all animals were negative at the pre-infection time point with PPD (B-A) and DST-P mean OD responses of − 0.12 ± 0.05 and 0 ± 0.06, respectively. Primary data are presented in Table S1.

**Table 1:** Overview of sampling schedules for immunology and microbiology.

	Weeks post infection									
	Pre	1	2	3	4	5	6	7	8	9
Experimental infection		X								
Immunology										
IGRA	X	X	X	X	X	X	X	X	X	
Serology	X	X	X	X	X	X	X	X	X	X
Skin test									X	
Microbiology										
Nasal swabs	X	X	X	X	X	X	X	X	X	X
Faecal samples	X	X	X	X	X	X	X	X	X	X
Necropsy										X

#### Tuberculin skin test

The raw data for the results of the tuberculin skin tests are detailed in supplemental Table S1. The tuberculin skin test was conducted at week 8 post-infection, one week prior to termination of the experiment and necropsy. Here, all animals tested positive to both cervical single intradermal tuberculin skin test (SIT) and comparative cervical tuberculin skin test (CCT), with the exception of one animal that did not cross the CCT interpretation of difference in skin thickness of > 4 mm. The mean (± standard deviation) increase in skin thickness observed in the SIT and CCT responses were 16 ± 7 mm and 13 ± 7 mm, respectively.

### Serological response in experimentally *M. bovis-*infected animals

**Figure 2:**
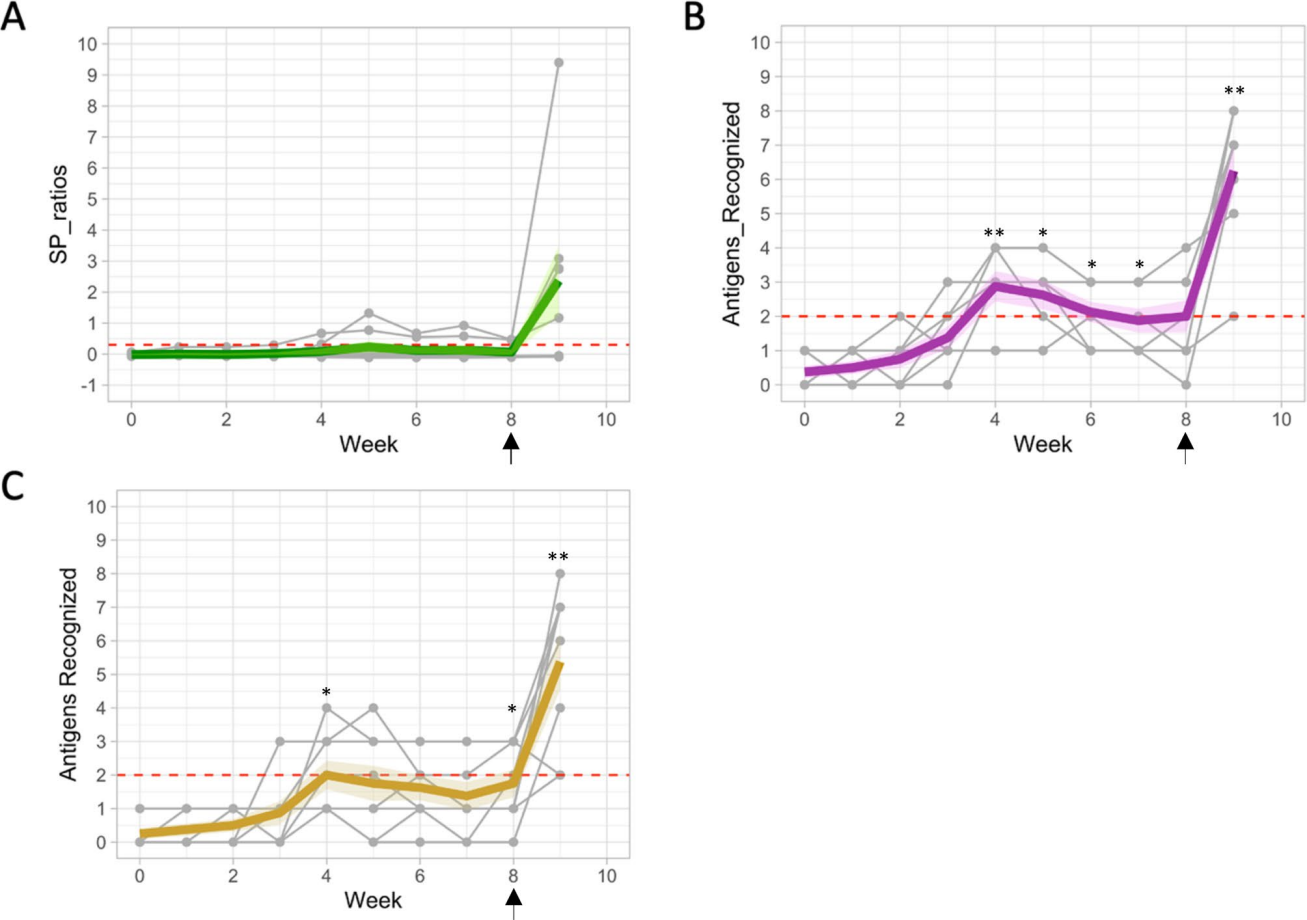
Serology response (*n* = 8). Blood samples from eight calves were taken both prior to and after experimental infection with *M. bovis* to isolate serum for use in IDEXX and Enfer commercial serological assays. Tuberculin skin test was conducted on week 8 (indicated by arrow). (**A**) IDEXX *M. bovis* assay; (**B**) Enferplex TB sensitivity optimized and (**C**) Enferplex TB specificity optimized settings. Data of individual animals are shown (thin grey lines) with trendlines (thick line) that display the sample mean (across animals) for each time point and 1 standard error on the mean (shaded area). Data is presented as (**A**) S/P ratio values or (**B** and **C**) number of test antigens recognized. Horizontal dashed red line: cut-off values for positivity. *p < 0.05, **p < 0.01, ANOVA with Dunnett’s multiple comparisons test (compared to the pre-challenge values).

Serological responses are summarised in Fig. 2A–C. Due to similar responses being observed between the animals, some of the lines for the individual animals merge in these graphs. However, for clarity, the raw data for each animal is summarized in Supplemental Table S1.

#### IDEXX *M. bovis* antibody ELISA

Humoral immune response post-infection was assessed using the commercially available IDEXX *M. bovis* Antibody ELISA kit. Serum samples were collected at weekly intervals (Table 1). It was observed that 2 of 8 animals showed positive antibody responses at week 4 post-infection and stayed positive for the rest of the study duration. A total of 5 of 8 animals were positive at the last time point post-skin test (week 9). At this time point (week 9), the mean (± standard deviation) antibody response elicited was found to be 2.4 ± 3.1 S/P ratios. Results are shown as S/P ratio where an S/P value of > 0.3 denotes a positive antibody response (Fig. 2A). Raw data are summarized in Table S1.

#### Enferplex TB antibody test

The Enferplex TB assay was performed at the same time points as indicated for the IDEXX *M. bovis* ELISA test (Table 1; Fig. 2). The assay was interpreted using two settings, namely a sensitivity or specificity optimized interpretation. For the high sensitivity interpretation of the test, lower cut-off values are applied for each of the test antigens compared with cut-off values for the high specificity interpretation. For either type of test interpretation, positive responses to 2 or more antigens were deemed positive. Prior to infection, none of the 8 animals tested positive when applying either high sensitivity or high specificity interpretations (Fig. 2B,C). As with IDEXX *M. bovis* test, application of a tuberculin skin test resulted in an anamnestic boost of the Enferplex TB responses such that all 8 animals tested positive one-week post-CCT. This result was independent of the interpretation criteria applied. The pre-CCT responses confirmed that the Enferplex TB test was able to detect a large proportion of infected animals prior to the CCT boosting. Applying the high sensitivity interpretation (Fig. 2B), positive test results were detected as early as 2 weeks post-infection (1 of 8 animals per the high sensitivity interpretation criteria), peaking between weeks 4 and 6 post-infection with either 4 of 8 or 6 of 8 calves testing positive with high specificity (Enfer Sp) or high sensitivity interpretations, respectively (Fig. 2B,C). We also observed fluctuation within individual animals between positive and negative test outcomes over the duration of the study period (Fig. 2B,C). Raw data are summarized in Table S1.

### Examination of nasal swabs and faecal samples for *M. bovis* shedding and confirmation of disease

Nasal swabs and faecal samples were collected weekly as indicated in Table 1. All samples underwent culture for *M. bovis* as well as being investigated by PCR*. M. bovis* could not be cultured from any of the nasal swabs, nor were positive PCR reactions observed (data not shown). However, we cultured *M. bovis* from one faecal sample collected from one animal at five weeks post-infection (animal 1638), but none were PCR-positive (data not shown). However, in an independent pilot study conducted with 4 animals to assess whether our sample collection protocol was well tolerated and did not lead to animal welfare issues (Table S2), we found two PCR-positive animals at one timepoint (pilot study, animals 1068 and 1078; data not shown).

Infection status was assessed by post-mortem examination and growth of *M. bovis* in culture. *M. bovis* was cultured from tissue samples collected from all animals. In addition, all animals demonstrated visible TB lesions in both lung and lymph node tissue. The extent of pathological changes was described using the scoring system of Vordermeier et al. ^13^, and the results are shown in supplemental Tables S1 and S3. For both the main study and the pilot study, there was no significant correlation between pathology scores and any of the CMI and humoral test results (data not shown).

**Figure 3:**
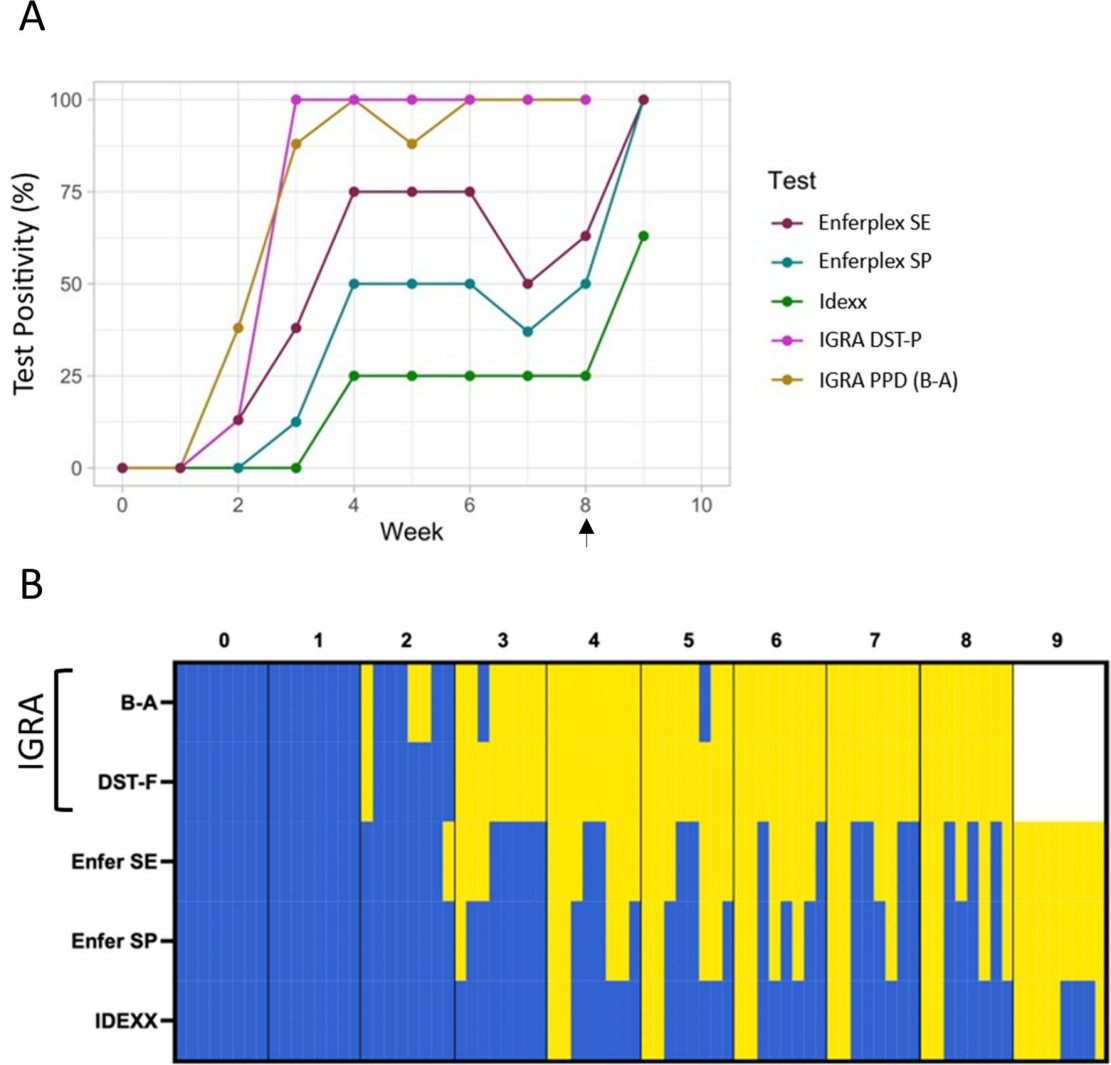
Summary of all CMI and serology-based tests conducted in the main study (*n* = 8 animals). (**A**) Positivity percentage at weekly intervals for all tests used is shown. (**B**) Binary heatmap showing individual animal trends in all tests used at weekly intervals. Each block defined by fine grey lines represents an animal. Blue represents ‘0’ or negative. Yellow represents ‘1’ or positive. Skin test was conducted at week 8 post-infection (indicated by arrow). Note that the last time point for IGRA was week 8, while serological assays were conducted until week 9 (one-week post-skin test). B-A: PPD(B-A) IGRA test; DST-P: DIVA skin test peptide cocktail IGRA; Enfer SE: high sensitivity interpretation of the Enferplex TB serology test; Enfer SP: high specificity interpretation of the Enferplex TB serology test; IDEXX: IDEXX serology test.

A summary of the temporal progression of early immune response against *M. bovis* infection in cattle is represented in Fig. 3. The outcomes of the tests shown in Fig. 3A were compared using Chi-Square statistics. Between week 2 (i.e. one week post infection) and week 8 (last timepoint prior to skin testing), all diagnostic tests were applied 64 times. Positive results were recorded 49, 49, 33, 20 and 10 times for IGRA B-A, IGRA DST-F, Enfer Se, Enfer Sp and IDEXX test, respectively. Thus significantly more positive results were obtained with both IGRA antigens compared to Enfer Se (p = 0.0033), Enfer Sp (p < 0.0001)) and IDEXX (p < 0.0001) serology tests. As shown in Fig. 3B, the majority of animals tested positive in IGRA prior to testing positive in the serological tests. The exception to this was one animal that tested positive to the high sensitivity interpretation of the Enferplex TB test at week 2 post infection. However, this animal did not test positive at the next time point, neither did it test positive to the high specificity interpretation of the Enferplex TB test or the IDEXX test at week 2 or 3 post infection. Although all animals that gave a positive test result to the IDEXX test also tested positive to both interpretations of the Enferplex TB test, there were no animals that only tested positive to the IDEXX test.

## Discussion

This study re-assessed the current model of the development of bovine immune responses to *M. bovis* infection using newer generations of sensitive and specific assays to detect CMI and serological responses. Current dogma is that CMI responses develop earlier and faster than humoral responses in *M. bovis* infected cattle ^14^, and that an anamnestic boost of the antibody responses following application of a tuberculin skin test enhances serum antibody responses ^15^. Therefore, assays such as the tuberculin skin test and associated blood tests that utilise the CMI response are to-date the mainstay of ante-mortem surveillance tests for bTB. However, the development of novel serological assays such as the Enferplex TB test warranted re-assessment of the dogma ^16^. Thus, we used experimentally infected calves to undertake a careful assessment of the temporal dynamics of the immune responses against tuberculous infection. The results (summarised in Fig. 3) confirmed that CMI, measured by IGRA, developed quickly post-infection with all 8 animals responding positively to stimulation with PPDs or the defined antigen formulation as early as 4 weeks post-infection. Humoral responses developed slightly later and required the anamnestic boost provided by application of a tuberculin skin test to reach comparable levels of diagnostic sensitivity for detection of infection (5 of 8 and 8 of 8 with IDEXX *M. bovis* and Enferplex TB assays, respectively). Interestingly, a proportion of animals developed responses prior to the immunological boost provided by the tuberculin skin test as measured by the IDEXX *M. bovis* and Enferplex TB serology tests. Particularly, application of the high sensitivity interpretation of the Enferplex TB assay detected 6 of 8 infected calves by week 4 post-infection. One animal in the pilot experiment (1 of 4) was also Enferplex TB test positive prior to infection applying either the high specificity or sensitivity optimized interpretation criteria (Supplementary information, Table S3). But this study was not designed to define test specificities, and due to the relatively small number of animals tested, it is not appropriate to make assumptions on relative test performances. The true proportion of animals developing detectable antibody titres and responding to serum tests prior to and independent of skin test boosting will have to be determined in field trials in different epidemiological settings. Interestingly, the level of anamnestic boosting post-tuberculin skin testing in individual animals was not associated with the degree of increase in skin thickness or the severity of pathology observed.

Although not observed in the main study, 2 of the animals in the pilot study reverted to IGRA test negative at the end of the study when using the differential readout of PPD-B minus PPD-A (Supplemental Table S3; Supplemental Fig. 1), despite showing visible signs of bTB pathology on post mortem examination. This appeared to be due to concomitantly high PPD-A responses that masked the PPD-B response. Encouragingly, these two animals generated a positive response to the defined antigen reagent DST-F at this time point, suggesting that this readout may be less impacted by cross-reactivity in IGRA. However, given the small number of animals tested in the studies presented herein, further test performance data comparing defined antigen reagents (e.g. DST-P or DST-F) to tuberculin reagents will be required to support changes to current IGRA test methodology.

We also assessed potential bacillary shedding routes by attempting to culture *M. bovis*, and detect its presence by PCR, in faecal and nasal swab samples. We could detect faecal shedding only in three animals on one occasion each, and nothing in the nasal swab samples. These observations suggest that while shedding of bacilli in faeces may occur even at relatively early time points post experimental infection, these are likely to be rare and paucibacillary events. While the small sample sizes preclude generalization, no differences in immune response trajectories were observed in these three animals compared to the rest of the infected calves (data not shown). The absence of culturable bacilli in nasal samples is not unexpected as our results are in line with the results of McCorry et al., who could detect bacillary shedding in nasal samples only following intranasal and very high dose intratracheal infection ^17^. At intratracheal infection doses comparable to the dose used in our two experiments, McCorry et al. did not observe *M. bovis* nasal shedding. It would be interesting to evaluate whether more frequent faecal or nasal shedding would occur at later stages post-infection. Unfortunately, extending the time period of our experiments was logistically and financially not possible. It is also noteworthy that there was no association with the three faecal shedding events and the trajectories of immune responses in these three animals. Although tests are available that promise to detect bacterial products in human TB sample matrices such as blood or urine, their utility for bovine TB is unproven, and nor is it clear whether detection of such markers is associated with the infectiousness of test-positive animals ^18–20^. It is also not clear whether a recently developed phage-based blood-borne *M. bovis* detection system can predict cattle at high risk of infectiousness ^21,22^. Therefore, it is important to perform rigorous studies of the trajectory of immune response and linking assessment of host responses and bacillary shedding to develop the next-generation of effective biomarkers that can identify animals at high risk of transmitting this disease.

In conclusion, this study assessed the temporal development of humoral and CMI responses after experimentally infecting cattle with *M. bovis*. Using state-of-the-art laboratory diagnostic tests, the results confirmed the temporal precedence of tests of CMI responses over those detecting humoral responses. The results also demonstrated that antibody responses may be detectable early post-infection, although early humoral responses have also been reported by Waters et al. ^9^, and independent of the so-called anamnestic tuberculin skin test boost. However, high sensitivities of the serological tests studied here still depended on skin test boost to match the sensitivity of IGRA. Taken together, our studies suggest that the current understanding of the trajectories of CMI and humoral immune responses post-*M. bovis* infection needs to be refined to include earlier serological responses than were previously predicted as per the dogma of binary CMI/humoral response kinetics in the original model. Of particular note, we find no discernible association between immune response trajectories and bacterial shedding, and our studies highlight an urgent need for research aimed at defining reliable biomarkers associated with bacterial shedding and infectiousness.

## Materials and methods

### Animals

*Bos taurus* calves were obtained from officially TB-free herds (Holstein-Friesians, males, 5–6 months old) and experimentally infected with *M. bovis* with 5000 CFU of a field strain (AF2122/97) via the endobronchial route ^13^. Two independent experiments were performed; a pilot experiment with 4 calves, followed by the main experiment with 8 calves using slightly modified methods in that animals were skin tested at 12 weeks post-challenge in the pilot trial as opposed to at 8 weeks in the main trial (Table 1; supplemental Table S2). Blood, faecal and nasal swab samples were collected at regular intervals (Table 1 and S2). Single intradermal comparative cervical tuberculin skin tests (SICCT or CCT) were performed as per the World Organization for Animal Health (WOAH, formerly “OIE”) manual using bovine and avian Purified protein Derivatives PPD (Thermo Fisher) at 3000 and 2000 IU per injection, respectively ^23^. At the end of the study, animals were killed by use of a captive bolt followed by destruction of the brain and exsanguination, and their infection status was confirmed by post-mortem and culture of *M. bovis*. The extent of pathological changes was described using the scoring system of Vordermeier et al. 2002. Details of the experimental and sampling schedules are shown in Tables 1 and S2. All animal procedures at APHA were approved by the APHA Animal Welfare and Ethical Review Board and conducted within the legal remit of a project licence under the Animals (Scientific Procedures) Act 1986, amended in 2013 granted by the UK Home Office. We confirm that the study is reported in accordance with ARRIVE guidelines, as applicable. All study methods were performed in accordance with the relevant guidelines and regulations.

### Antigens

Bovine and Avian Purified Protein derivatives (PPD-B and PPD-A) were purchased from Thermo Fisher, UK. The DIVA skin test (DST) fusion protein consisting of the mycobacterial antigens ESAT-6, CFP-10 and Rv3615c (DST-F) was obtained from Lionex (Braunschweig, Germany). A peptide cocktail of DST composed of 13 overlapping peptides covering the amino acid sequences of the same three antigens (DST-P) ^24^ was purchased as individual peptides from Genscript. Pokeweed mitogen (PWM) was obtained from Sigma, UK. All antigens were diluted in RPMI-1640 when used for in vitro whole blood stimulations. Due to reagent availability, DST-P was used in the experiment with 8 animals while DST-F in the pilot trial with 4 animals.

### In vitro stimulation of whole blood

Heparinised blood samples were stored overnight at room temperature before stimulation (250 µl in duplicate wells of a 96-well plate) for 20 to 24 h at 37 °C in 5% CO_2_ with DST-F (1 µg/ml), DST-P (1 µg/ml), PPD-B (300U/ml) and PPD-A (250 U/ml), Pokeweed Mitogen (5 µg/ml; PWM, positive control) and RPMI-1640 medium alone (negative control). After stimulation, blood was centrifuged at 300 g for 10 min and the plasma supernatant was harvested and stored at − 80 °C until required.

### Interferon-gamma release assay (IGRA)

IFN-γ in plasma culture supernatants was quantified using the commercially available BOVIGAM enzyme-linked immunosorbent assay (ELISA) kit (Thermo Fisher Scientific, USA). IGRA results for DST-P stimulated blood cultures were expressed as the optical density at 450 nm (OD_450_) for cultures stimulated with DST-P minus the OD_450_ for cultures without antigen (i.e., ΔOD_450_) with a ΔOD_450_ > 0.1 defined as test positive. Comparative PPD-A and PPD-B responses are shown as OD_450_ (PPD-B minus PPD-A); test positivity was defined as OD_450_ PPD-B minus PPD-A > 0.1. Positive and negative control data are not shown.

### Skin test procedure

Skin tests were carried out as previously described ^23^*.* Injection sites located in the border of the anterior and middle third of the neck on either side of the cow were clipped and skin thickness recorded. PPD-A and PPD‑B were administered in a 0.1 ml volume via intradermal injection as per the manufacturer’s recommendations (PPD-B: 3000 U/dose, PPD-A: 2500 U/dose). Skin thickness was measured again by the same operator 72 h after administration, and the difference in skin thickness (mm) between the pre- and post-skin test readings recorded. For the single intradermal tuberculin skin test, greater than or equal to 4 mm increase in skin thickness was considered a positive reaction. For the comparative cervical tuberculin skin test, greater than 4 mm increase in skin thickness was considered a positive reaction.

### Serology

#### IDEXX *M. bovis* antibody test

The test was performed as per manufacturer’s instructions (IDEXX Laboratories Ltd) as previously described ^25^. Briefly, serum samples were diluted 1:50 using kit sample diluent before being transferred to the test plate in duplicate. Plates were incubated at room temperature (RT) for 1 h before washing and addition of kit conjugate. After a 30-min incubation at RT, plates were washed again before addition of kit TMB substrate. Stop solution was added after a 15-min incubation period at RT and plates were read at 450 nm using a Multiskan FC plate reader (Thermo Scientific) per manufacturer’s instructions. Samples are presented as an S/P ratio where the delta OD values of the samples are divided by the delta OD value of the internal kit positive control. Sample is deemed positive if the S/P ratio is 0.30 or greater.

#### Enferplex TB serology test

The Enferplex TB serology test is a multiplex chemiluminescent immunoassay developed to simultaneously detect antibody recognition of multiple antigens in a single well ^16^. A proprietary collection of 11 antigens consisting of soluble products, recombinant proteins either singly or in mixtures or as fusion proteins, and synthetic peptides were selected based on reactions detected using negative and positive sera from animals known to be free from or infected with *M. bovis*. The antigens were deposited in a multiplex planar array as individual 30 nl spots into wells of 96 well black polystyrene microtiter plates using BioDot (Irvine, CA, USA) aspirate/dispense platforms per manufacturer’s instructions. Plates were blocked, stabilised, dried and stored at 2–8 °C until use. Serum samples were diluted 1:200 into sample dilution buffer (Enfer Buffer B, Enfer Scientific) and mixed before 50ul was added per well. The plates were incubated at 37 °C for 60 min with agitation (900 rpm). The plates were washed 6 times with 1 × Wash (Enfer Wash buffer, Enfer Scientific) and aspirated. The detection antibody, sheep anti-bovine IgG—HRP (Bethyl Laboratories) was prepared to 1:20,000 dilution. The plates were incubated at 37 °C for 60 min with agitation (900 rpm). The plates were washed as above and 50ul of prepared chemiluminescent substrate (1:1 ratio of substrate and diluent, (Advasta, USA) was added per well. Relative light units (RLU) were captured (220 s exposure) immediately, using Quansys Biosciences Q-View™ LS imager and Q-View™ software (v 3.1.15). The results were defined using the Enferplex Bovine TB Macro developed by the manufacturer, based on individual antigen thresholds after subtracting the RLU value obtained from a blank spot. The individual antigen thresholds were set using known positive and negative serum samples. The Enferplex Bovine TB Macro uses proprietary knowledge to set two different cut-off values which were applied to either a sensitivity or specificity optimized interpretation criteria. A positive response is defined as having responses above these cut-off points for at least two antigens (spots).

#### Faecal samples

Faecal samples were collected by insertion of a collection tube into the rectum of the animals. 2 g of faeces were placed in 20 ml of 0.75% 1-Hexadecylpyridin-1-ium chloride, vortexed vigorously for 10 s, and left to stand for 30 min. Then 10 ml of the supernatant was transferred to a clean 30 ml universal centrifuge tube and left to stand overnight at room temperature. The sample was centrifuged at 3000*g* for 15 min, the supernatant was discarded, and the pellet was re-suspended in 2 ml of sterile PBS pH 7.2. One MGIT BBL tube was prepared by adding 0.8 ml of MGIT960 supplement (Becton Dickinson) and was inoculated with 500ul of the sample then placed in the MGIT 960 instrument for 42 days after which any growth was further examined by Kinyoun staining. A further three Modified 7H11 slopes were inoculated with 300ul of the sample each and incubated at 37 °C for 12 weeks. Agar plates were examined after 4–6 weeks and after 12 weeks incubation for *M. bovis* colony growth.

#### Nasal swab samples

Nasal swab samples were collected using Swabs procured from Newcaombe Medical, Newmarket, UK (SE16/S/L/5000). Samples were immersed in 4 ml sterile PBS pH 7.2 at collection and then stored at − 70 °C. Immediately before culture, samples were thawed for one hour at room temperature, vortexed for ten seconds and the swabs removed from the PBS and discarded. The samples were then centrifuged at 3200 × *g* for 15 min at room temperature and the supernatants discarded. 0.5 ml sterile PBS pH 7.2 was then added to each sample before vortexing again for a further 10 s. Two Modified 7H11 agar plates were then each inoculated with 50 µl of each sample and incubated at 37 °C for a total of 12 weeks. Agar plates were examined after 4–6 weeks and after 12 weeks incubation for *M. bovis* colony growth.

#### *M. bovis* PCR

A single tube RT-nPCR was used to target specific mycobacterial sequences to confirm the presence of *M. bovis*. All steps to undertake the single, closed tube reaction used a modification of the protocols of Wolff et al. and McGoldrick et al. ^26,27^. Nested PCR primers and dual-labelled probe for the second stage reaction of the nested PCR were dried and stabilized into the lids of the eight well PCR strips of lids used to seal the PCR reactions. Strips of lids were prepared by adding 8 μL of liquid containing 5 μL 20% (w/v) trehalose (Sigma, Gillingham, UK), 1 μL 20 µmol nested primer forward (NPF), 1 μL of 20 µmol nested primer reverse (NPR), and 1 μL of 5 µmol probe to each lid and allowing to air dry in a Class I biological safety cabinet. Once dry, strips of lids could be stored in the dark at room temperature in plastic bags. For the IS1081 assay, the NPF was 5′ CTGCTCTCGACGTTCATCGCCG 3′, the NPR was 5′ TGGCGGTAGCCGTTGCGC 3′, and the probe [HEX] was 5′ ATTGGACCGCTCATCGCTGCGTTCGC 3′ [black hole quencher 1]. For the RD4 assay, the NPF was 5′ TGTGAATTCATACAAGCCGTAGTCG 3′, the NPR was 5′ ATGGCTATTGACCAGCTAAGATATCCG 3′, and the probe [CY5] was 5′ CAACACTCTTGGAGTGGCCTACAACGGC 3′ [Black Hole Quencher 2]. PCR reactions for the first stage of the nested PCR were prepared using the QuantiTect Multiplex PCR No ROX Kit (Qiagen, Manchester, UK) and dispensed into 20 μL aliquots (in 96-well PCR plates), to which 5 μL of template DNA was added. Concentrations in the final PCR were: 1 × master mix and 0.8 µmol of both outer primer forward (OPF) and outer primer reverse (OPR). For IS1081, OPF was 5′ TGGCTGACCAACTCGCACAGGC 3′ and OPR was 5′ TCGCGACGTCGATGGTTGCGGCAC 3′. For RD4, OPF was 5′ AATGGTTTGGTCATGACGCCTTCC 3′ and OPR was 5′ CCCGTAGCGTTACTGAGAAATTGC 3′. Reactions were sealed with the strips of caps containing the dried reagents for the matching assay prepared as described above. Reactions were cycled on an MX3000P PCR instrument as follows: 95 °C for 5 min; 20 cycles of 95 °C for 30 s, 60 °C for 30 s, and 72 °C for 30 s. No fluorescent measurements were taken. The PCR plate was then removed and inverted to allow the liquid from the PCR reactions to dissolve the nested PCR reagents dried into the lids. Following a brief centrifugation step to settle the contents of the tubes, the PCR plate was added back into the real-time PCR instrument and cycled as follows: 95 °C for 5 min; 40 cycles of 95 °C for 30 s, 60 °C for 30 s, and 72 °C for 30 s. Fluorescent measurements were taken at the end of each 60 °C step.

#### Post mortem examination

For each animal, the lungs and the following lymph nodes were removed and examined: right and left bronchial; cranial mediastinal; caudal mediastinal; and cranial tracheobronchial. The severity of TB pathology was quantified using a scoring system previously described ^13^.

#### Statistical analysis

Data was analyzed using GraphPad Prism and R Studio software. R Studio was used for the descriptive analysis (trend line and 1 standard error on the mean) shown in Figs. 1 and 2. Data in Figs. 1 and 2 was analysed by ANOVA with Dunnett’s multiple comparisons test, with post infection time points compared to the pre-challenge (week 0) values. Data in Fig. 3 was analysed using Chi-Square test, with a Bonferroni correction for multiple comparison error applied using a significance level of 0.0088 (significance level of 0.05/6 comparisons).

### Supplementary Information

Supplementary Information 1.

Supplementary Tables.

## Data Availability

All data needed to evaluate the conclusions in the paper are present in the paper and/or the Supplementary Materials.
