# Supplementary material for: Temporal dynamics of the early immune response following Mycobacterium bovis infection of cattle
Source: Sci Rep. 2024 Jan 31;14:2600. doi: 10.1038/s41598-024-52314-x (PMC10831113; doi:10.1038/s41598-024-52314-x)
Supplement: Supplementary file 1 — Supplementary Information 1. [file 41598_2024_52314_MOESM1_ESM.docx]

**SUPPLEMENTAL INFORMATION**

**Supplemental Table 1.** Raw data of main experiment.

**Supplemental Table 2.** Pilot trial schedule.

**Supplemental Table 3.** Raw data of pilot experiment.

**Supplemental information: Additional methods**

**Pilot experiment Results**

A pilot experiment was conducted mainly to confirm the practicability and animal welfare-compatibility of our intensive sampling protocols (Table S2). We also took the opportunity to generate initial immunological data. Four Holstein-Friesians (males, 5-6 months old) were experimentally infected with *M. bovis* with 5,000 CFU of a field strain (AF2122/97) via the endobronchial route. Blood, faecal and nasal swab samples were collected regularly (Table S2). Skin test was conducted at week 12 in accordance with the OIE manual, one week prior to necropsy. Details of the experimental and sampling schedules are shown in Table S2. No adverse effects due to this sampling schedule were observed and we therefore implemented an identical one in the main experiment.

**IGRA.** The PPDs and DST-F (a recombinant fusion protein, DSTF, comprising of antigens ESAT-6, CFP-10, Rv3615c) were used for stimulation *in vitro.* Animals started showing interferon-gamma responses as early as 2 weeks post-infection. All animals crossed the BOVIGAM cut-off of 0.1 for both PPD(B-A) and DSTF as early as week 4 post-infection and remained positive for the rest of the trial duration, with the exception of two animals that dropped below cut-off for PPD(B-A) at week 13 (post-skin test). At (week 4 post-infection), the animals elicited a mean (± standard deviation) IFN-γ response to PPD (B-A) and DST-F of 1.5±0.9 and 2.4±1.8, respectively. The PPD (B-A) and DST-F responses attained peaks at weeks 8 and 14 with mean responses of 2.5±0.7 and 4.5±0.6, respectively. Importantly all animals remained negative at the pre-infection time point with PPD (B-A) and DST-F mean responses of -0.29±0.53 and 0.01±0.0, indicating high specificity of both PPD(B-A) and DST-F used in the interferon gamma release assay (Figure S1).

**Skin test.** Skin test was conducted once towards the end of the experimental period, just prior to necropsy (Table S2). All four animals tested positive to both SIT and CCT with mean (± standard deviation) responses of 21±10 and 17±8, respectively (Table S2).

**IDEXX *M. bovis* Antibody ELISA.** Serum samples were collected at weekly intervals (Table S2). The antibody responses were undetectable at all time points prior to the skin test. However, at one-week post-skin test, all animals (*n* = 4) showed antibody responses. At this time point (week 13), the mean (± standard deviation) antibody response elicited was found to be 1.6±1.4 (Figure S1).

**Enferplex TB Antibody test.** Prior to infection, 1/4 animal tested positive when applying either high sensitivity or high specificity interpretations (Figure S1). When we considered responses prior to CCT application and disregarding the false-positive animal, 2/3 animals tested positive by week 4, (high specificity interpretation), although results for these two calves tended to move between positive and negative test outcomes. Applying the high sensitivity interpretation did not alter the positivity rates. Fluctuations within individual animals between positive and negative test outcomes were also observed (Figure S1, Table S3). One week after applying the tuberculin skin test, all animals (4/4) tested positive regardless of whether high sensitivity or high specificity settings were applied (Figure S1).


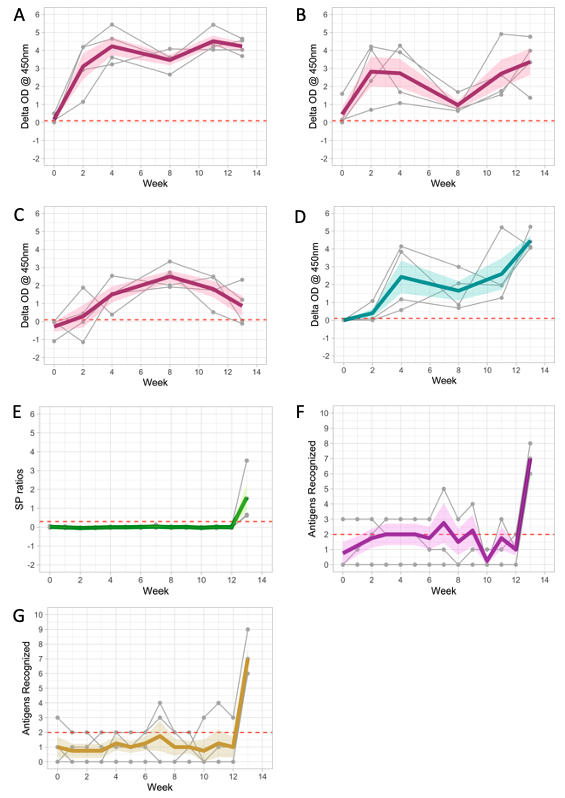


**Supplementary Figure 1. CMI- and serology-based responses (*n* = 4).** Blood samples from four calves were taken both prior to and after experimental infection with *M. bovis* for use in whole blood IGRA using tuberculin and DST-F reagents and for isolation of serum for use in *M. bovis* serology tests. The background-corrected (delta) optical density (OD) values are plotted for IGRA. Time is shown as weeks post-infection in the x-axis. Skin test was conducted at week 12 post-infection. Data of individual animals are presented (thin lines) with thick lines indicating trend lines and 95% CIs. A: PPD-B; B: PPD-A; C: PPD(B-A); D: DST-F; E: IDEXX *M. bovis* assay; F: Enferplex TB sensitivity optimized and G: Enferplex TB specificity optimized settings.
